# Lipoxin A4 levels predict site-specific clinical improvements post scaling and root planing and correlate negatively with periodontal pathogens in severe periodontitis

**DOI:** 10.1186/s12903-024-03948-w

**Published:** 2024-02-08

**Authors:** Rui Ma, Yiying Liu, Yi Xu, Dingyu Duan

**Affiliations:** 1 State Key Laboratory of Oral Diseases, Department of Periodontics, West China Hospital of Stomatology, National Clinical Research Center for Oral Diseases, Sichuan University, No. 14, Section 3, Renmin South Road, Chengdu, 610041 China; 2 Department of Stomatology, The First Affiliated Hospital of Zhengzhou University, Zhengzhou, 450052 China

**Keywords:** Lipoxins, Periodontitis, Prognosis, Root planing, Gingival crevicular fluid

## Abstract

**Background:**

Serving as a stop signal of inflammation, the role of lipoxin A4 (LXA4) in periodontitis remains to be clarified. This study is aimed to examine the changes in LXA4 levels in gingival crevicular fluid (GCF) after scaling and root planing (SRP) and to determine the relationship between LXA4 levels and treatment outcomes and periodontal pathogens in severe periodontitis.

**Methods:**

A total of 74 GCF samples were collected from 21 severe periodontitis participants at the deepest affected sites. These sites were re-sampled at 1, 3, and 6 months after SRP. Besides, GCF samples were also collected from 25 periodontally healthy participants. Clinical parameters including probing depth (PD) and clinical attachment level (CAL) in periodontitis group were recorded. LXA4 levels and periodontal pathogens in the GCF were analyzed by ELISA and PCR, respectively. Correlations between GCF LXA4 levels and treatment effect and periodontal pathogens were assessed.

**Results:**

LXA4 levels in GCF significantly increased after SRP (*p* < 0.05), but remained lower than those observed in healthy individuals (*p* < 0.05). Sites with lower baseline LXA4 concentrations were more likely to experience greater improvements in PD at 6 months post-SRP (area under the curve [AUC] = 0.792), and the improvements were positively correlated with the increase of LXA4 at these sites post-treatment (*p* < 0.05). Furthermore, more elevated LXA4 levels were observed in sites that became negative for *Prevotella intermedia* or *Tannerella forsythia* after SRP.

**Conclusion:**

Baseline LXA4 in GCF has the potential to predict the site-specific response of severe periodontal lesions to SRP. The increase of LXA4 levels after treatment was positively correlated with clinical improvements and negatively correlated with the presence of *Prevotella intermedia* or *Tannerella forsythia*.

**Supplementary Information:**

The online version contains supplementary material available at 10.1186/s12903-024-03948-w.

## Introduction

Periodontitis is a chronic multifactorial inflammatory disease, characterized by microbially-associated, host-mediated inflammation that results in loss of periodontal attachment and, ultimately, loss of tooth [1, 2]. Given the high prevalence and systemic implications of periodontitis, research into the pathogenesis and treatment of periodontal disease is crucial for enhancing oral and overall health outcomes [3–5].

Periodontal pathogens, including *Porphyromonas gingivalis (P. gingivalis)*, *Aggregatibacter actinomycetemcomitans (A. actinomycetemcomitans)* and etc., constitute a necessary etiological factor for the initiation of periodontitis [6]. These pathogens elicit excessive immune-inflammatory responses which can exacerbate the destruction of periodontal tissues [7]. Current data suggest that the excessive inflammation in periodontitis is associated with a failure in inflammation resolution [8, 9]. Resolution of inflammation has been demonstrated to be actively orchestrated by a unique group specialized proresolving mediators (SPMs), which are derived from essential polyunsaturated fatty acids, including lipoxins (LXs), resolvins, protectins, and maresins [10–12]. Accumulating evidence has established the ability of SPMs to limit neutrophil infiltration, reduce tissue damage, clear microbial infections, and participate in stem cell proliferation and differentiation [11, 13, 14].

Serving as a stop signal of inflammation [15, 16], LXs, including LXA4, LXB4, and aspirin-triggered LXs, are generated from arachidonic acid by interactions between individual lipoxygenases [10]. Pre-clinical models have demonstrated the potential and efficacy of LXA4 in preventing the onset of periodontitis, and in treating periodontal diseases by regenerating lost periodontium [17–19]. However, research regarding the changes in LXA4 secretion by individuals in a clinical setting is scarce.

Despite the documented efficacy of scaling and root planing (SRP) in reducing substantial levels of periodontal inflammation [20], the complete suppression of inflammation remains a significant challenge. As a result, the development of adjunctive methods that can be effectively combined with mechanical debridement to manage periodontitis, particularly in severe cases, is imperative. Given LXA4 is a molecule with potential for future clinical application in promoting resolution of inflammation and tissue repair, elucidating the changes in LXA4 levels during states of periodontal health and periodontitis, as well as after periodontal treatment can help us understand its role in the development and alleviation of periodontitis.

As far as we are aware, only a limited number of cross-sectional studies have explored the levels of LXA4 in human gingival crevicular fluid (GCF) or saliva [21–23]. To date, there is a dearth of longitudinal studies that examine the relationship between LXA4 levels and the therapeutic efficacy of periodontal treatment. Besides, due to the different susceptibilities to periodontal diseases and different responses in specific sites, individualizing periodontal care is important [24]. GCF is the promising medium to reflect more precise information in specific sites [25]. Therefore, in this study, we aimed to examine the changes in GCF LXA4 levels after SRP treatment in patients with severe periodontitis, and explore the relationship between LXA4 and treatment outcomes and periodontal pathogens. Finally, based on these experimental results, we assessed the ability of baseline LXA4 to predict site-specific treatment effects of SRP.

## Materials and methods

### Study participants

Participants were recruited from the Department of Periodontology, West China Hospital of Stomatology, Sichuan University from 2018 to 2021. The study protocol was approved by the Ethics Committee of West China Hospital of Stomatology (Number: WCHSIRB-D-2017-092) and was conducted in accordance with the Declaration of Helsinki in 2013. Written informed consent was obtained from all study participants. All participants were systemically healthy, as confirmed by physical examination and a comprehensive blood examination. The inclusion criteria for the severe periodontitis group were as follows: (i) at least 20 natural teeth, including at least 12 premolars and molars (excluding third molars); and (ii) at least five sites with probing depth (PD) and clinical attachment loss/level (CAL) ≥ 6 mm at baseline in different quadrants. A full-mouth series of X-rays within the past 6 months were required to further assess the diagnosis of periodontitis. All patients with periodontitis recruited met the criteria for a diagnosis of severe periodontitis [26], and they also need to be diagnosed with Stage III/IV periodontitis according to the 2018 classification of periodontitis [1]. Healthy controls had no periodontal sites with attachment loss or PD > 3 mm, and a whole mouth BOP positive site count of < 10%.

The exclusion criteria were as follows: (i) pregnancy or lactation; (ii) known medical disorders that could affect local and systemic inflammatory status and the cytokine levels in oral fluids, such as diabetes mellitus, rheumatoid arthritis, and immunological disorders; (iii) treatment with any anti-inflammatory drugs or antibiotics in the previous three months; (iv) history of periodontal treatment in the previous six months; (v) past or current smokers; (vi) significant occlusal disharmony or a history of orthodontic treatment in the past 10 years; and (vii) an inability to provide consent.

### Sample size calculation

Changes of GCF LXA4 levels in periodontitis after treatment were used to calculate the optimal sample size. Due to a lack of related information in the literature, a priori sample calculation could not be performed. Thus, a pilot study which included five periodontitis patients was performed, and the changes in LXA4 levels before and after treatment were recorded. Sample size was determined by a function of power and level of significance. A sample size of 20 in the periodontitis group was required to detect a significant difference of 10 pg/µl of LXA4 with 80% statistical power and a 5% level of significance. If the sample size is calculated based on the detection of changes of PD, only about 6 patients are needed to achieve 90% statistical power and a 5% level of significance. Therefore, we chose a sample size of 20 to meet the requirement for statistical testing of both PD and LXA4.

### Clinical assessment

After demographic factors were recorded, all participants underwent a full-mouth periodontal examination by the same examiner (RM). This examiner was trained in a calibration process to reduce intra-examiner error [27]. Five periodontitis patients were chosen for calibration. PD was measured on two occasions, 24 h apart. For PD, the percentage of agreement within ± 1 mm between repeated measurements was at least 95%. PD was measured from the free gingival margin to the base of the pocket. CAL was calculated as PD plus gingival recession. PD and CAL were recorded to the nearest millimeter at six sites per tooth (mesio-buccal, mid-buccal, disto-buccal, disto-lingual, mid-lingual, and mesio-lingual), except for the third molars, with a UNC-15 probe (Hu-Friedy, Chicago, IL, USA ). BOP was recorded as present or absent after 30 s of PD measurements. The BOP percentage was calculated by dividing the number of bleeding sites by the total number of sites examined in each participant. The plaque score (PS) was recorded as the presence or absence of plaque [28]. Additionally, PD reduction (≥ 2 mm) and CAL gain (≥ 3 mm) are regarded as the primary treatment outcome measures.

### Clinical intervention

At the initial visit, periodontal measurements of all participants were recorded, and their baseline GCF samples were collected at least two days later. Patients with severe periodontitis underwent oral hygiene instructions, full-mouth supragingival debridement, and quadrant-based SRP using both ultrasonic scalers (MiniPiezon, EMS Dental, Nyon, Switzerland) and hand instruments (Gracey Curette; Hu-Friedy, Chicago, IL, USA) under local anesthesia within one month. Follow-up examinations and GCF sample collections were conducted at 1, 3, and 6 months after treatment, with clinical measurements taken at each subsequent follow-up appointment. In addition, supportive periodontal therapy including oral hygiene instructions and supragingival debridement was provided at each subsequent visit.

### GCF sampling

In both groups, two to four sites per individual were selected as the sampling sites and GCF samples were collected by a single calibrated investigator (RM). In the healthy group, GCF samples were collected from sites with no clinical inflammation. With regard to patients with severe periodontitis, GCF samples were collected from each individual at the deepest sites (PD and CAL ≥ 6 mm) of their mouth.

After isolation of the sampling sites with cotton rolls to prevent the contamination of saliva, large and visible supragingival plaques were removed with Gracey curettes without touching the marginal gingiva. The sampling site was gently air-dried, and a 30-second GCF sample was collected using a filter paper strip (3MM, Whatman, Kent, UK), which was gently inserted into the sulcus/pocket, 1–2 mm subgingivally [29]. Samples visibly contaminated with blood or saliva were discarded and then collected from another site. Every filter paper strip was placed in a sterile Eppendorf tube (Axygen, Corning Incorporated, NY, USA) that had been weighed before sampling, using an analytical balance (AE 240s, Mettler, Zurich, Switzerland) with a sensitivity of 0.01 mg, and reweighed immediately after sampling. The increase of the weights was used to calculate the volume of GCF, according to relationship between weight and volume of GCF reported [30, 31]. These samples were stored at -80 °C until use. Eventually, 64 GCF samples from periodontally healthy participants and 74 GCF samples from severe periodontitis participants were collected in total, which were all individually used for biochemical and microbiological analysis.

### Analysis of LXA4 in GCF

GCF samples were analyzed by an enzyme-linked immunosorbent assay (ELISA). Before the quantification of LXA4 levels, 300 µl phosphate-buffered saline containing 0.25% bovine serum albumin was added to each Eppendorf tube, and then the samples were centrifuged for 60 min at 300 rpm (8 g) and then for 2 min at 12,000 rpm (13,201 g) at a temperature of 4 °C [29]. The supernatants were collected and LXA4 levels were estimated using a commercially available ELISA kit (Neogen Corp., Lansing, MI, USA ) in accordance with the manufacturer’s instructions.

The diluted GCF samples were placed in duplicate in two wells. The total amount (pg) of LXA4 per sample in a 30 s period was identified using standard curves according to the manufacturer’s guidelines and dilution factor [29], and then divided by the GCF volume to give the LXA4 concentration (pg/µl) in the original GCF sample. Two values were obtained for each sample and the average of these values was taken as the final figure.

### Analysis of periodontal pathogens

The precipitate of the pretreatment GCF sample from patients with severe periodontitis at baseline and 6 months after treatment was used for bacterial DNA extraction. DNA was extracted using an DNA extraction kit (Tiangen Biotech, Beijing, China) according to the manufacturer’s instructions. The bacterial 16 S ribosomal DNA of *P. gingivalis*, *A. actinomycetemcomitans*, *Treponema denticola (T. denticola)*, *Prevotella intermedia (P. intermedia)* and *Tannerella forsythia (T. forsythia)* were amplified by polymerase chain reaction (PCR) system (Bio-Rad, California, USA ), using primers as previously reported [32, 33]. Genomic DNA from the plasmid-generated standards or reference strains were used as the positive control and distilled water was used as the negative control. PCR products were separated by electrophoresis in 2% agarose gels.

### Statistical analysis

Statistical analysis was performed using the statistical software program SPSS version 21.0 (IBM Corp., Armonk, NY, USA ) and MedCalc version 19.0, and *p*-value < 0.05 was considered statistically significant. Graphs were created using GraphPad Prism version 7.0 (GraphPad Software Inc., San Diego, CA, USA ). Results based on continuous data were presented as mean ± SD, and results based on categorical data were presented as numbers and percentages.

Normal distribution of the data at baseline was checked using the Shapiro-Wilk test. Since the data was found to be non-normally distributed, the variables were analyzed with non-parametric methods. First, when examining group differences in demographic and clinical characteristics at baseline, the Pearson’s chi-square (χ2) test was used for categorical variables, whereas the non-parametric Mann-Whitney U were applied to determine differences in continuous data. Second, comparisons of the clinical parameters and the LXA4 levels before and after treatment in the severe periodontitis group were analyzed using the Wilcoxon signed rank test. Third, given that different GCF sampling sites within a patient could be more correlated with each other than the sites between patients and independent variables like gender, age and oral hygiene could potentially have relevant effects on the outcomes, we used generalized estimating equation (GEE) models adjusting for these variables to examine the relationship between LXA4 levels and clinical parameters (PD reduction ≥ 2 mm or CAL gain ≥ 3 mm) in this longitudinal study. The discrimination of the predicting model was assessed with receiver operating characteristic (ROC) analyses and the area under the curve (AUC). The optimal criterion for each ROC analysis was identified according to Youden’s index. Additionally, GEE analysis was also used to identify relationships between LXA4 levels and specific periodontal pathogens. Note that two patients with severe periodontitis were unavailable for analysis at the 1-month and 3-month follow-up visits, respectively.

## Results

### Demographic and clinical characteristics

A total of 48 subjects were screened for inclusion in this study. Of these subjects, 2 were excluded due to unavailability of contact. Eventually, 25 periodontally healthy people and 21 patients with severe periodontitis were included in the analysis (Figure S1). According to the 2018 classification of periodontitis [1], 17 patients were diagnosed with Stage III, 4 patients were diagnosed with Stage IV, and all patients were classified as Grade C.

The demographic and clinical characteristics of the participants from all study groups examined at baseline are presented in Table 1. No significant differences were observed between groups in terms of gender (*p* > 0.05), but the mean age of patients with severe periodontitis was significantly higher than that of periodontally healthy participants (*p* < 0.05).

**Table 1:** Demographic characteristics and clinical parameters of study groups

	Healthy	Severe periodontitis
	**(n = 25)**	**(n = 21)**
Gender(male/female)	8/17	9/12
Age (years)*	22.00 ± 2.40	37.48 ± 9.85^a^
Teeth number*	30.08 ± 1.50	30.43 ± 1.69
PS (%)*	27.69 ± 7.01	65.67 ± 13.15^a^
BOP (%)*	8.64 ± 0.82	75.92 ± 13.42^a^
PD in the whole mouth(mm)*	1.90 ± 0.72	4.69 ± 0.92^a^
PD in sampling sites(mm)*	2.11 ± 0.65	7.69 ± 1.37^a^
CAL in the whole mouth (mm)*	1.90 ± 0.72	4.99 ± 1.00^a^
CAL in sampling sites(mm)*	2.11 ± 0.65	7.78 ± 1.40^a^
Percentage of teeth with pockets ≥ 5 mm*	-	88.20 ± 13.74
Percentage of sites with pockets ≥ 5 mm*	-	50.28 ± 17.98
Percentage of teeth with ≥ 33% bone loss*	-	42.44 ± 26.86
Percentage of teeth with ≥ 50% bone loss*	-	29.44 ± 27.13
Periodontitis Stage^#^ III IV	- -	17 (81.0) 4 (19.0)
Periodontitis Grade^#^ C	-	21(100.0)

Abbreviations: BOP, bleeding on probing; PD, probing depth; CAL, clinical attachment level

*Values are given as means ± standard deviation

#Values are given as n (%) of participants

a, *p* < 0.05, compared with healthy controls

At baseline, there was no significant difference in total number of teeth between groups (*p* < 0.05), but the percentage of PS and BOP of periodontitis participants was markedly higher than those of periodontally healthy group (*p* < 0.05) (Table 1). Clinical parameters of patients with severe periodontitis before and after SRP are shown in Table S1. Significant improvements were observed in PD and CAL scores for the whole mouth and individual sampling sites at 1, 3, and 6 months post-treatment when compared to baseline measurements (*p* < 0.05). The percentages of BOP and PS also showed a remarkably decrease following SRP (*p* < 0.05).

### Effect of SRP on LXA4 levels in GCF

As shown in Table 2, baseline LXA4 concentration was significantly lower in the severe periodontitis group compared to the healthy control group (86.13 ± 60.81 pg/µl vs. 251.46 ± 128.13 pg/µl, *p* < 0.05). The concentration of LXA4 in the periodontitis group showed a statistically significant increase compared to baseline at the 1-month, 3-month, and 6-month follow-up visits post-treatment. The mean concentration of LXA4 in periodontitis increased from a low of 86.13 ± 60.81 pg/µl at baseline to a high of 232.16 ± 317.86 pg/µl at 3 months post-therapy. However, it always remained statistically lower than baseline concentration in the healthy control group (*p* < 0.05).

**Table 2:** Concentrations and total amounts of GCF LXA4 in the study groups before and after SRP

	Healthy (n = 64)	Severe periodontitis (n = 74)
**LXA4 concentrations ** **(pg/µl)**		
Baseline	251.46 ± 128.13	86.13 ± 60.81^a^
1 m	-	163.09 ± 159.45^a,b^
3 m	-	232.16 ± 317.86^a,b^
6 m	-	139.20 ± 107.11^a,c^
**LXA4 total amounts ** **(pg/sample)**		
Baseline	59.38 ± 22.08	42.69 ± 22.85^a^
1 m	-	58.19 ± 30.20^b^
3 m	-	62.69 ± 37.22^b^
6 m	-	57.27 ± 31.27^b^
**GCF volume ** **(µl)**		
Baseline	0.30 ± 0.13	0.56 ± 0.22^a^
1 m	-	0.49 ± 0.24^a,b^
3 m	-	0.44 ± 0.23^a,b^
6 m	-	0.53 ± 0.24^a^

Two patients with periodontitis were unavailable for the 1month and 3 months follow-up respectively

Abbreviations: m, month

a, *p* < 0.05, compared with healthy controls at baseline;

b, *p* < 0.05, compared with periodontitis group at baseline;

c, *p* < 0.05, compared with periodontitis group at 3 months after SRP;

SRP resulted in a reduction in the volume of GCF in the periodontitis group (Table 2), and the total amount of LXA4 showed different trends compared to its concentration. In the severe periodontitis group, the total amount of LXA4 increased after therapy, ranging from 42.69 ± 22.85 pg/sample to 62.69 ± 37.22 pg/sample, with the highest levels observed at 3 months post-treatment. Moreover, the total amounts of LXA4 in the periodontitis group at all follow-up time points after treatment were not significantly different from those in the healthy group (*p* > 0.05).

### Association between LXA4 and clinical parameters in severe periodontitis

In severe periodontitis, GCF samples were collected from each individual at the deepest sites of their mouth, where PD and CAL values were equal to or greater than 6 mm (with a range of 6-11 mm). Although at the patient level, severe periodontitis group had lower levels of LXA4 than healthy group, at the site level of severe periodontitis, the baseline LXA4 concentrations and total amounts were not correlated with the baseline PD and CAL at these deep sites (Table S2).

Data derived from the GEE models evaluating the relationship between LXA4 concentrations and treatment effect in severe periodontitis, adjusted for gender, age, and plaque, are provided in Tables 3, 4 and 5. As shown in Table 3, sites with greater improvements in PD at 3 and 6 months post-treatment tended to have lower baseline LXA4 concentrations (*p* < 0.05). Meanwhile, greater increases in LXA4 concentrations post-treatment were observed at these sites. Despite the greater increase, the LXA4 concentrations at these sites at 3- or 6-months post-treatment were not significantly higher compared to those at other sites. As for CAL improvement, there was no significant difference in LXA4 concentrations between groups (Table S3).

**Table 3:** The levels of LXA4 at different sites with varying degrees of PD reduction at different timepoints after SRP in patients with severe periodontitis were analyzed after adjusting for gender, age, and plaque accumulation

**Concentrations (pg/µl)**	ΔPD ≥ 2 mm at 1 m			ΔPD ≥ 2 mm at 3 m			ΔPD ≥ 2 mm at 6 m		
	**Yes ** (***n* = 52)**	**No ** (***n* = 18)**	***p***	**Yes ** (***n* = 52)**	**No ** (***n* = 14)**	***p***	**Yes ** (***n* = 59)**	**No ** (***n* = 15)**	***p***
Baseline LXA4	82.53 ± 56.11	99.41 ± 75.45	0.251	77.91 ± 56.92	109.22 ± 77.53	0.017	79.51 ± 54.80	112.16 ± 77.00	0.018
LXA4 post-treatment	172.59 ± 163.20	135.63 ± 149.03	0.408	248.30 ± 351.89	172.22 ± 120.36	0.497	148.80 ± 114.39	101.44 ± 60.72	0.189
Increase of LXA4 post-treatment	90.06 ± 166.12	36.22 ± 103.75	0.148	170.40 ± 347.38	63.00 ± 115.82	0.294	69.29 ± 113.98	-10.72 ± 70.84	0.017

Values are given as mean ± SD

Abbreviations: m, month; ΔPD, PD reduction from baseline to corresponding post-treatment

Our results demonstrated that sites with lower LXA4 concentrations at baseline tended to exhibit a greater increase in LXA4 levels at 6 months after SRP (Table S4). Besides, GEE analysis further revealed that sites with a greater increase in LXA4 concentration had a higher probability of achieving more improvements in PD (OR = 2.684, 95%CI 1.189–6.057) at 6 months post-treatment (Table 4).

**Table 4:** Relationships between the increase in LXA4 concentration and future PD improvements in patients with severe periodontitis after adjusting for gender, age and plaque accumulation

LXA4 increase in concentrations (per 100 pg/µl)	ΔPD ≥ 2 mm at 3 m		ΔPD ≥ 2 mm at 6 m	
	OR (95%CI)	***p***-value	OR (95%CI)	***p***-value
3 m	1.193 (0.858, 1.660)	0.294	**-**	-
6 m	**-**	**-**	2.684 (1.189, 6.057)	0.017

Abbreviations: m, month; ΔPD, PD reduction from baseline to the indicated time point after SRP

ΔCAL, CAL gain from baseline to the indicated time point after SRP

OR (95% CI), odds ratio (95% confidence interval)

**Fig. 1:**
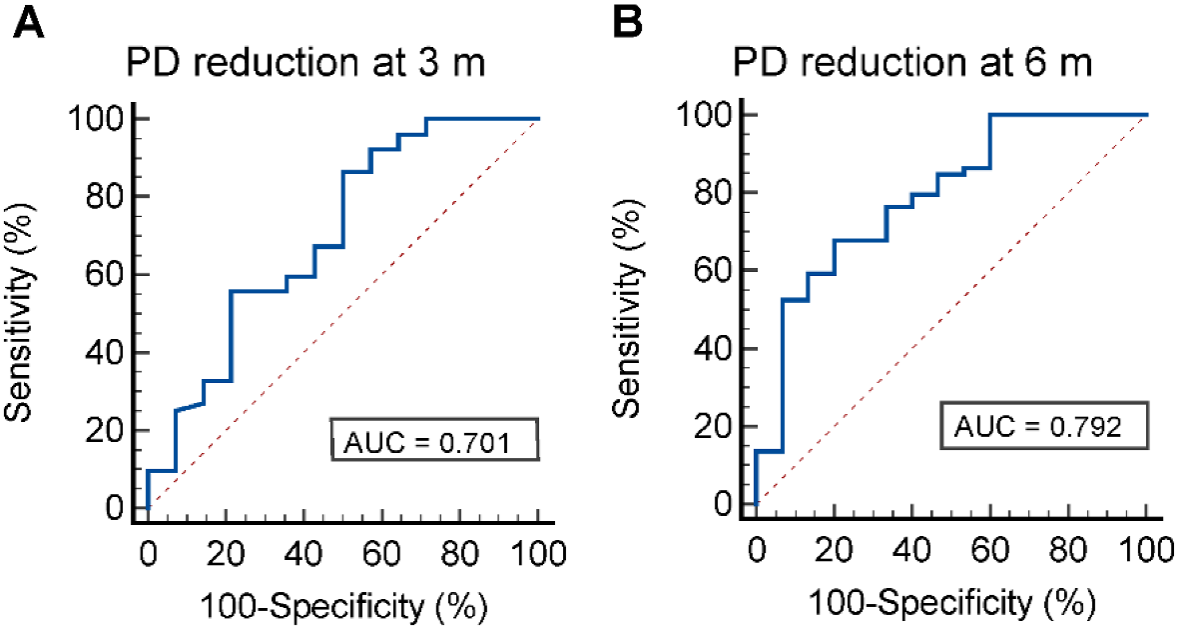
ROC curves were constructed to evaluate the predictive ability of baseline LXA4 concentrations, as determined by the GEE model, for the treatment outcomes of SRP in severe periodontitis patients. The ROC curves were generated to assess the ability of baseline LXA4 concentrations to predict (**A**) PD reduction (≥ 2 mm) at 3 months after SRP. (**B**) PD reduction (≥ 2 mm) at 6 months after SRP. PD, probing depth

Results from Table 5 showed that sites with lower baseline LXA4 concentrations had a higher likelihood of exhibiting more improvements in PD at 6 months after SRP (OR = 0.327). Similar findings were observed for the reduction of PD at 3 months after SRP (OR = 0.419). Moreover, the receiver operating characteristic curve analysis showed that the model predicting PD reduction at 6 months after SRP using baseline LXA4 concentrations had a larger AUC of 0.792 (95%CI 0.682–0.878), compared to the model predicting PD reduction at 3 months after SRP with an AUC of 0.701 (95%CI 0.576–0.808) (Table 5; Fig. 1).

**Table 5:** The predictive role of baseline LXA4 concentrations on PD improvements after SRP in patients with severe periodontitis analyzed by adjusted GEE models

	Baseline LXA4 concentrations(per 100 pg/µl)						
	**OR ** **(95%CI)**	***p*** **-value**	**QIC**	**AUC ** **(95%CI)**	**Cut-off**	**Sensitivity**	**Specificity**
ΔPD ≥ 2 mm at 3 m	0.419 (0.197, 0.894)	0.024	67.2	0.701 (0.576, 0.808)	0.7	86.50%	50%
ΔPD ≥ 2 mm at 6 m	0.327 (0.127, 0.839)	0.02	67.65	0.792 (0.682, 0.878)	0.84	67.80%	80%

Abbreviations: m, month; ΔPD, PD reduction from baseline to the indicated time point after SRP

OR (95% CI), odds ratio (95% confidence interval)

QIC, Quasi-likelihood under the independence model criterion

### Associations between LXA4 and the presence of subgingival bacteria

**Fig. 2:**
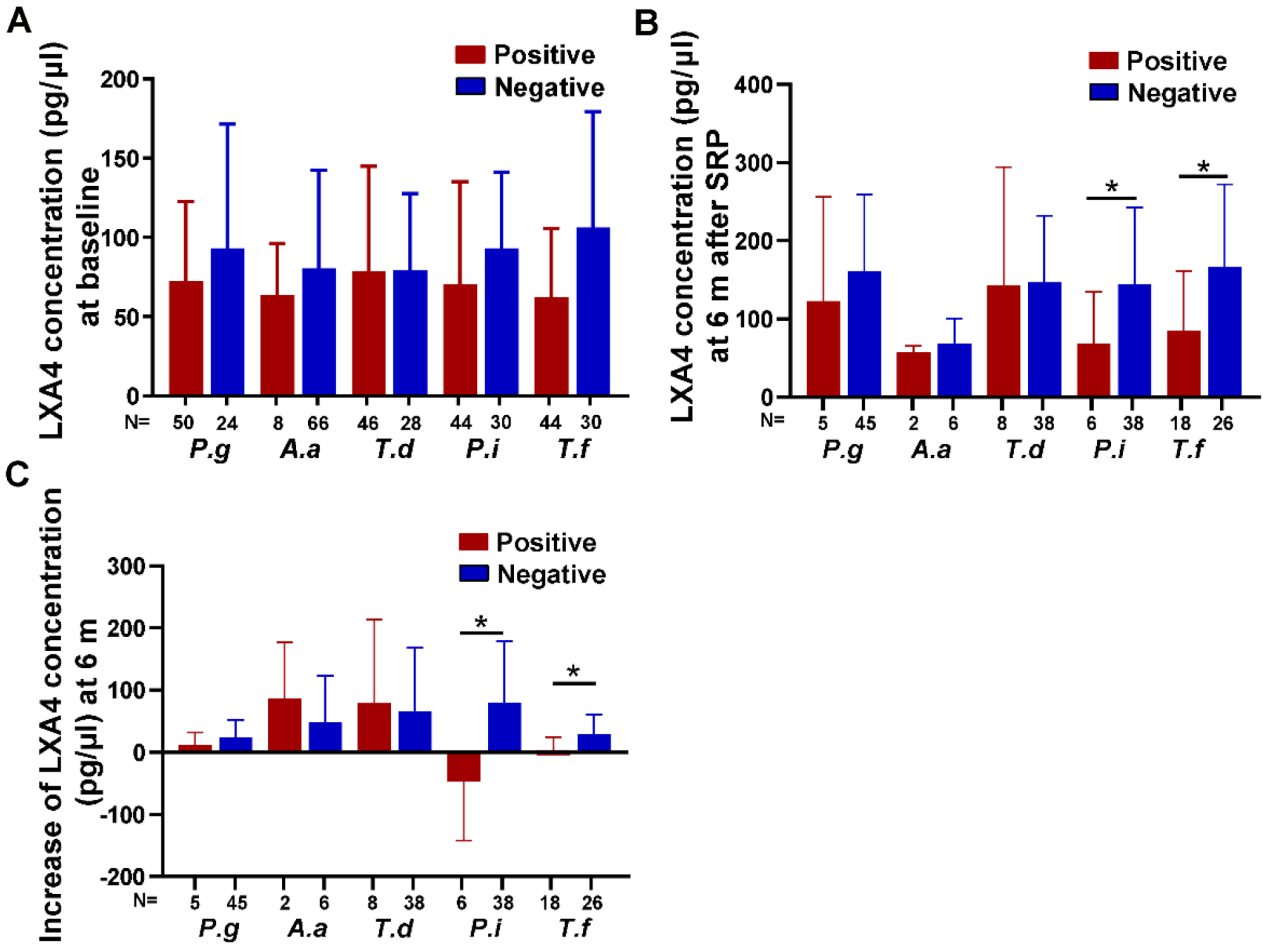
Comparison of LXA4 concentrations in GCF between sites that were positive or negative for periodontal pathogens at (**A**) baseline, (**B**) 6 months after SRP and (**C**) the increase in LXA4 concentration between sites that were still populated with presumed microorganisms or not at 6 months after treatment. Significant difference between groups are represented as * (*p* < 0.05). N, number of sites; *P.g*, *Porphyromonas gingivalis*; *A.a*, *Aggregatibacter actinomycetemcomitans*; *T.d*, *Treponema denticola*; *P.i*, *Prevotella intermedia*; *T.f*, *Tannerella forsythia*

A total of 74 sites were eventually involved in microbial analysis. At baseline, *P. gingivalis* was detected in 50 of the 74 sites, *A. actinomycetemcomitans* was detected in only 8 sites, *T. denticola* was detected in 46 sites, and *P. intermedia* and *T. forsythia* were detected in 44 sites. Although the decrease of LXA4 concentration in these periodontal pathogens positive sites could be observed, the differences were not statistically significant (*p* > 0.05) (Fig. 2A).

Of the 50 sites positive for *P. gingivalis*, 5 remained positive 6 months after treatment, while 2 of the 8 sites positive for *A. actinomycetemcomitans*, 6 of the 44 sites positive for *P. intermedia*, 8 of the 46 sites positive for *T. denticola*, and 18 of the 44 sites positive for *T. forsythia* remained positive. LXA4 concentration was statistically lower in *P. intermedia* or *T. forsythia* positive sites compared with the negative sites at 6 months post-treatment (*p* < 0.05) (Fig. 2B). Additionally, the increase in LXA4 concentration was lower in sites where *P. intermedia* or *T. forsythia* remained positive compared with the sites that became negative at 6 months post-treatment (*p* < 0.05) (Fig. 2C).

## Discussion

This study found that the GCF level of LXA4 in patients with severe periodontitis significantly increased after SRP, but remained lower than the levels observed in healthy individuals. In order to compare our results with previous studies [21–23], we focused on the LXA4 concentrations in the subsequent analysis. The results showed that deep sites (PD and CAL ≥ 6 mm) with lower baseline LXA4 concentrations had a higher probability for more PD improvements at 6 months after SRP in patients with severe periodontitis, and the improvements were positively correlated with the increase of LXA4 at these sites post-treatment. This study also observed a negative correlation between LXA4 concentration increase and the presence of certain periodontal pathogens post-treatment.

SRP has been considered the cornerstone of cause-related therapy, but it does not necessarily result in significant clinical improvement in all subjects or all sites in the same subject, especially in cases of advanced stages and with deep periodontal pockets [34, 35]. This was also confirmed in our study where 15 out of a total of 74 sites (all of which were deep pockets at baseline) had a PD reduction of no more than 1 mm at 6 months after SRP. Therefore, identifying those poorly responded diseased sites will contribute to the precision periodontal treatment. A recent study showed that periodontitis patients were associated with a disturbance in salivary SPMs [23]. Although saliva is readily available and non-invasive as a diagnostic fluid, it could only reflect information about the whole mouth of the patient without accurate information about specific periodontal sites. Therefore, this study employed GCF as a sample source, as it can be easily obtained in proximity to the affected periodontal tissues and contains rich molecular information [29].

In this study, we selected PD reduction ≥ 2 mm and CAL gain ≥ 3 mm after SRP as the primary outcome variables in subsequent analysis as previously described [27, 36–40]. Dividing the sites into different groups according to the PD and CAL threshold changes instead of calculating mean changes of these clinical parameters was considered to be reasonable and preferable in evaluating treatment effect [37].

The current study found that patients with severe periodontitis had significantly lower baseline LXA4 concentrations in GCF compared to healthy controls, and SRP did not result in LXA4 concentrations being restored to the levels seen in healthy controls. This suggests that a decrease in LXA4 concentration may serve as an indicator of destructive periodontal inflammation, which is consistent with most of the previous research findings [22, 23]. By contrast, Lutfioglu et al. found that the GCF LXA4 concentrations in periodontally healthy individuals were below the detection threshold [21]. The limited diffusion of GCF in healthy gingiva [41] makes the sampling of GCF in periodontally healthy sites more challenging and may account for the discrepancies observed in periodontally healthy participants.

Our findings revealed an association between lower baseline LXA4 concentrations and greater improvements in PD during follow-up assessments after SRP. This indicates that, at baseline, prior to the elimination of most of the bacteria through SRP, high levels of LXA4 in the presence of a high bacterial load in periodontal tissue may not be of benefit to the host. Furthermore, our study demonstrated that at sites with lower baseline LXA4 concentrations, post-treatment increases in LXA4 were significantly larger and positively correlated with clinical improvement. These results suggest that the regulation of LXA4 concentrations, including decreases and increases, needs to be carefully modulated according to the immune state, so as to trigger the protective immune response or reduce inflammation as appropriate. In this study, no significant difference was found between baseline LXA4 concentrations and CAL improvements after SRP. We proposed that maybe CAL gain is harder to achieve compared to PD reduction and in most cases its improvement would be by recession.

From current studies, LXA4 was considered to inhibit the chemotaxis and adhesion of neutrophils, and promote macrophages to uptake apoptotic neutrophils in a non-phlogistic manner [42, 43]. A recent clinical trial reported that an oral rinse containing a stable analog mimetic of lipoxin A4 effectively reduced gingival inflammation [44]. Our research also supports the role of LXA4 in reducing inflammation, but further indicates that the stage of tissue homeostasis remodeling after initial periodontal treatment may be the best time for LXA4 to play a role.

In the light of the negative association between lower baseline LXA4 concentrations and follow-up clinical improvements, we evaluated the ability of baseline LXA4 concentrations to predict PD improvements at 3 and 6 months after SRP. Collectively, baseline LXA4 concentrations had an acceptable discrimination ability for PD levels at 6 months after SRP (AUC = 0.792). Different patients and different sites do not necessarily respond equally to initial periodontal treatment [24]. Identifying the poorly responded sites is of great clinical importance. This information could aid in risk assessment and precise treatment planning for periodontal disease.

Periodontitis manifests as a destructive inflammatory response of the periodontal tissues to a complex biofilm composed of bacteria and their byproducts [41]. To date, the relationship between lipoxins and subgingival periodontal pathogens remains unknown. Therefore, in this study, LXA4 levels and periodontal pathogens in the same GCF sample collected by filter paper strips were analyzed. The detection rate of *A. actinomycetemcomitans* in patients with periodontitis was consistent with previous reports [45, 46]. However, probably due to different sampling or processing methods, the detection rate of other periodontal pathogens in GCF at baseline and 6 months after therapy was lower compared with previous studies [30, 47].

At the 6-month follow-up, our study found that the sites where *P. intermedia* or *T. forsythia* became negative had significantly higher LXA4 concentrations compared to sites that remained positive for these pathogens. Furthermore, the increase in LXA4 levels was also found to be higher in these sites. LXA4 has the capacity to activate monocytes into a non-phlogistic phenotype, which could enhance the clearance of bacteria [10, 11]. This may be one of the reasons for the negative correlation between LXA4 and certain periodontal pathogens after treatment. Certainly, it is also reasonable to assume that these periodontal pathogens, particularly *P. intermedia* and *T. forsythia*, may affect the production or degradation of LXA4, leading to a decrease in local LXA4 levels and a prolonged inflammation. Further studies are needed to elucidate the underlying mechanisms.

Although significant findings were identified, this study still has some limitations. Large-scale populations are needed to better understand the role of LXA4 in the pathogenesis and healing process of periodontal diseases. To further explore the etiological role of LXA4, a prospective cohort study is needed to observe the incidence of periodontal disease in populations with varying levels of LXA4. Additionally, it should be noted that GCF analysis may not provide comprehensive information about periodontal pathogens, as compared to direct sampling of subgingival plaque. Nonetheless, this study sheds light on LXA4 alterations following SRP and explores its potential as a predictive marker for site responses to SRP in GCF.

## Conclusion

The present study was first to explore LXA4 alterations after SRP in severe periodontitis patients and relationships between LXA4 and treatment outcomes and periodontal pathogens. Baseline LXA4 in GCF has the potential to predict the site-specific response of severe periodontal lesions to SRP. The increase of LXA4 levels after treatment was positively correlated with the clinical improvements and negatively correlated with the presence of *P. intermedia* or *T. forsythia*. Our results extend our understanding of LXA4 and provide a potential biological factor for future clinical risk assessment.

### Electronic supplementary material

Below is the link to the electronic supplementary material.

Supplementary Material 1

## Data Availability

The datasets used during the current study are available from the corresponding author on reasonable request.
