# Supplementary material for: Lipoxin A4 levels predict site-specific clinical improvements post scaling and root planing and correlate negatively with periodontal pathogens in severe periodontitis
Source: BMC Oral Health. 2024 Feb 8;24:204. doi: 10.1186/s12903-024-03948-w (PMC10851498; doi:10.1186/s12903-024-03948-w)
Supplement: Supplementary file 1 — Supplementary Material 1 [file 12903_2024_3948_MOESM1_ESM.docx]

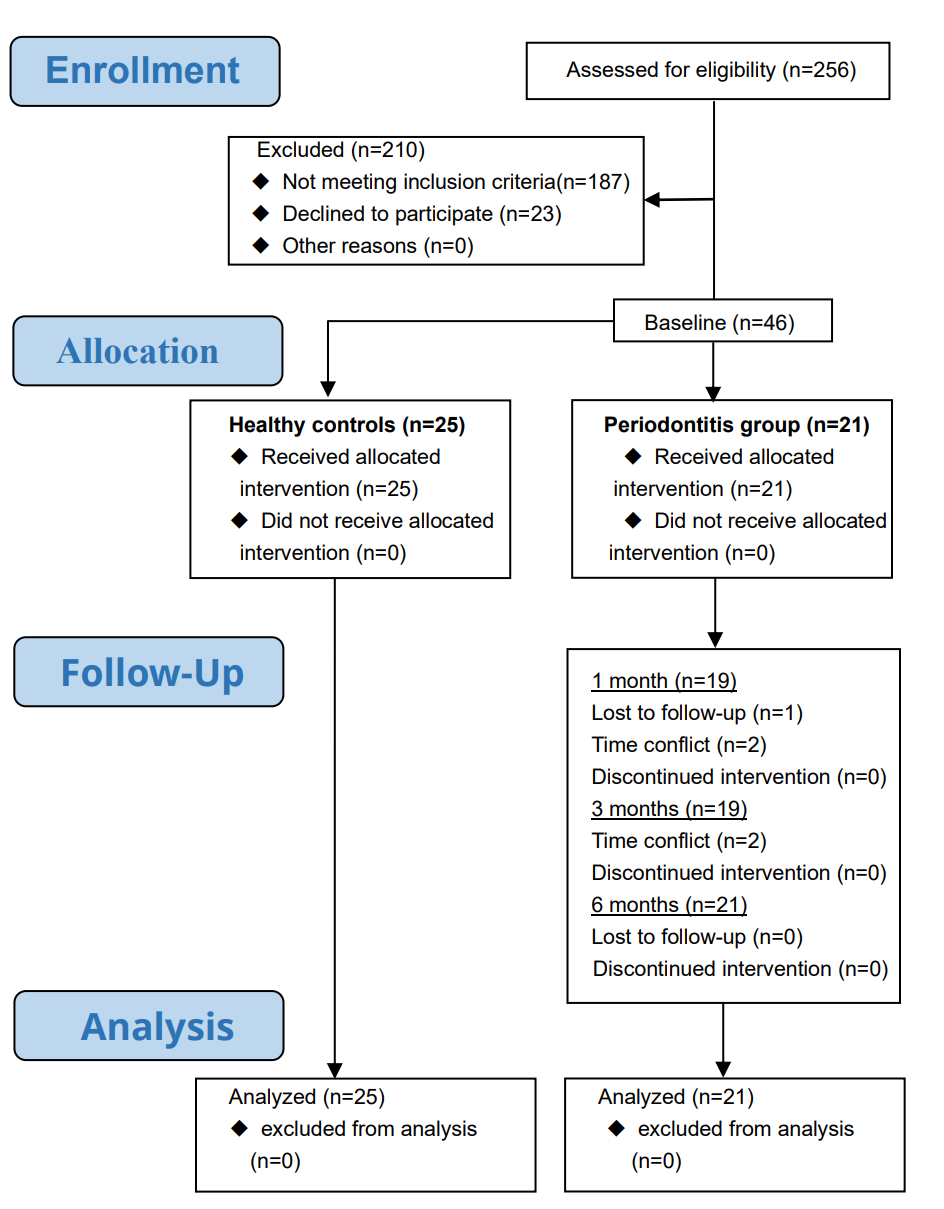


Figure S1. Flow chart of the study.

**Table S1. Clinical parameters of patients with severe periodontitis at different examination timepoints**

|  | **Baseline** | **1 m** | **3 m** | **6 m** |
| --- | --- | --- | --- | --- |
| **Whole mouth** |  |  |  |  |
|  | (n = 21) | (n = 19) | (n = 19) | (n = 21) |
| PD (mm)* | 4.69±0.92 | 3.63±0.60^a^ | 3.46±0.55^a^ | 3.32±0.49^a^ |
| CAL (mm)* | 4.99±1.00 | 4.23±0.99^a^ | 4.10±0.95^a^ | 3.97±0.87^a^ |
| BOP (%) * | 75.92±13.42 | 28.89±10.38^a^ | 29.70±8.91^a^ | 25.88±10.62^a^ |
| PS (%)* | 65.67±13.15 | 36.57±12.67^a^ | 37.00±15.45^a^ | 31.88±19.72^a^ |
| **Sampling sites** |  |  |  |  |
|  | (n = 74) | (n = 70) | (n = 66) | (n = 74) |
| PD (mm)* | 7.69±1.37 | 5.21±1.58^a^ | 4.89±1.72^a^ | 4.82±1.73^a^ |
| CAL (mm)* | 7.78±1.40 | 5.73±1.83^a^ | 5.36±2.05^a^ | 5.35±1.94^a^ |
| BOP^#^ | 70(94.59) | 30(42.86) | 34(51.52) | 41(55.41) |
| PS^#^ | 59(79.73) | 42(60.00) | 28(42.42) | 29(39.19) |

* Values are given as means ± standard deviation

# Values are given as n (%) of participants.

a, *p*＜0.05, compared with baseline.

Abbreviations:

m, month; PD, probing depth; CAL, clinical attachment level; BOP, bleeding on probing

**Table S2 Relationships between LXA4 levels and periodontal destructions after adjusting for gender, age and plaque accumulation in severe periodontitis**

|  | **Baseline LXA4 concentrations  (pg/μl)** | | **Baseline LXA4 total amounts (pg/sample)** | |
| --- | --- | --- | --- | --- |
|  | OR (95%CI) | *p*-value | OR  (95%CI) | *p*-value |
| Baseline PD | 0.998  (0.995, 1.001) | 0.126 | 1 (0.990, 1.011) | 0.988 |
| Baseline CAL | 0.999  (0.996, 1.001) | 0.279 | 1.001  (0.992, 1.010) | 0.875 |

Abbreviations: OR (95% CI), odds ratio (95% confidence interval);

PD, probing depth; CAL, clinical attachment level

**Table S3 The levels of LXA4 at different sites with varying degrees of CAL gain at different timepoints after SRP in patients with severe periodontitis were analyzed after adjusting for gender, age, and plaque accumulation**

|  | **△CAL≥3mm at 1 m** | | | **△CAL≥3mm at 3 m** | | | **△CAL≥3mm at 6 m** | | |
| --- | --- | --- | --- | --- | --- | --- | --- | --- | --- |
| **Concentrations (pg/μl)** | Yes （n = 30） | No  (n = 40) | *p* | Yes (n = 35) | No (n = 31) | *p* | Yes (n = 39) | No  (n = 35) | *p* |
| **Baseline LXA4** | 85.33±10.19 | 88.03±10.46 | 0.921 | 79.35±9.84 | 90.41±12.14 | 0.452 | 74.72±8.87 | 98.85±10.95 | 0.187 |
|  |  |  |  |  |  |  |  |  |  |
| **LXA4 post-treatment** | 186.12±35.54 | 145.81±20.05 | 0.123 | 293.13±69.23 | 163.34±25.11 | 0.389 | 159.32±19.66 | 116.79±13.94 | 0.131 |
| **Increase of LXA4 post-treatment** | 100.80±35.41 | 57.78±18.02 | 0.114 | 213.77±68.93 | 72.92±22.61 | 0.326 | **84.60±19.16** | **17.94±15.19** | **0.005** |

Values are given as mean ± SD.

Abbreviations: m, month; △CAL, CAL gain from baseline to corresponding post-treatment

**Table S4 Relationships between baseline LXA4 levels and future increase in LXA4 levels after SRP in patients with severe periodontitis after adjusting for gender, age and plaque accumulation**

|  | **Baseline LXA4 concentrations （pg/μl）** | |  |
| --- | --- | --- | --- |
|  | OR (95%CI) | *p*-value |  |
| Increase in concentrations at 1 m | 4.103  (0.066, 253) | 0.502 |  |
| Increase in concentrations at 3 m | 0.732  (0.394, 1.358) | 0.323 |  |
|  |  |  |  |
| Increase in concentrations at 6 m | **0.566  (0.450, 0.711)** | **＜0.001** |  |
|  |  |  |  |

Abbreviations: m, month; OR (95% CI), odds ratio (95% confidence interval)
